# Supplementary material for: Clinical Significance of the Plasma Biomarker Panels in Amyloid-Negative and Tau PET-Positive Amnestic Patients: Comparisons with Alzheimer’s Disease and Unimpaired Cognitive Controls
Source: Int J Mol Sci. 2024 May 21;25(11):5607. doi: 10.3390/ijms25115607 (PMC11171590; doi:10.3390/ijms25115607)
Supplement: Supplementary file 1 [file ijms-25-05607-s001.zip › ijms-2986083-supplementary.pdf]

Clinical Significance of Plasma Biomarkers Panel in Amyloid-Negative and Tau PET Positive Amnestic Patients: Comparisons with Alzheimer's Disease and Cognitive Unimpaired Controls

Supplementary Materials

Table S1: Descriptive Statistics of 90 cognitive unimpaired controls (CTL)

	Total Tau (pg/ml)	NFL (pg/ml)	pTau181 (pg/ml)	Aβ42/40ratio	Aβ42 (pg/ml)	Aβ40 (pg/ml)	Age_at_plasma
Median	4.072	11.412	1.538	0.045	9.066	200.907	67.000
Mean	4.309	14.757	1.812	0.045	9.325	214.801	65.229
95% CI Mean Upper	4.551	16.926	2.113	0.047	9.869	230.377	67.199
95% CI Mean Lower	4.066	12.912	1.571	0.043	8.813	201.650	63.197
Std. Deviation	1.277	10.320	1.330	0.010	2.621	74.165	10.028
IQR	1.254	7.648	0.928	0.013	2.360	78.536	12.000
Shapiro-Wilk	0.928	0.713	0.634	0.982	0.907	0.857	0.940
P-value of Shapiro-Wilk	5.310e-5	2.119e-12	4.111e-14	0.213	4.529e-6	3.525e-8	2.810e-4
Minimum	1.373	4.412	0.100	0.014	2.499	58.079	25.000
Maximum	8.393	77.036	11.420	0.071	22.080	554.445	88.000

Plasma samples analyzed using single-molecule array analysis, with units pg/ml; IQR interquartile range
NFL: neurofilament light chain. One CTL group showed extreme NFL data is excluded.

Table S2: Descriptive Statistics of 151 Alzheimer's disease (AD) patients

	Total Tau (pg/ml)	NFL (pg/ml)	pTau181 (pg/ml)	Ab42/40ratio	Aβ42 (pg/ml)	Aβ40 (pg/ml)	Age_at_plasma
Median	5.108	21.655	3.326	0.037	8.081	220.152	72.000
Mean	6.054	28.739	3.652	0.038	8.280	224.291	72.623
95% CI Mean Upper	7.383	32.642	3.995	0.039	8.734	236.418	73.934
95% CI Mean Lower	5.190	25.179	3.297	0.036	7.864	212.058	71.391
Std. Deviation	7.273	25.885	2.157	0.008	2.720	78.914	7.955
IQR	2.628	17.305	1.970	0.008	2.833	88.036	10.000
Shapiro-Wilk	0.269	0.557	0.810	0.979	0.973	0.953	0.992
P-value of Shapiro-Wilk	5.048e-24	1.708e-19	1.452e-12	0.023	0.005	6.363e-5	0.565
Minimum	1.654	5.737	0.050	0.010	0.883	48.033	46.000
Maximum	88.947	254.261	15.049	0.064	17.140	580.923	94.000
Fold changes (CTL as reference)	1.25	1.90	2.16	0.82	0.89	1.09	NA
Fold changes (TCP as reference)	1.21	1.25	1.89	0.90	0.84	0.93	NA

One case of YOAD had extremely high total tau (88.95 pg/ml) and 2 cases with extremely high NFL (129.32 and 254.27) and were excluded from the analysis for the rest comparisons.

Table S3: Descriptive Statistics of 44 Tau first cognitive proteinopathy (TCP) patients

	Total Tau (pg/ml)	NFL (pg/ml)	pTau181 (pg/ml)	Aβ42/40ratio	Aβ42 (pg/ml)	Aβ40 (pg/ml)	Age_at_plasma
Median	4.233	17.287	1.760	0.041	9.583	237.844	75.500
Mean	11.303	29.503	2.353	0.042	9.890	247.911	73.295
95% CI Mean Upper	24.334	48.232	2.935	0.046	11.140	280.880	75.796
95% CI Mean Lower	4.392	18.233	1.835	0.039	8.847	219.740	70.841
Std. Deviation	42.186	53.427	1.919	0.013	3.912	98.210	8.741
IQR	1.816	17.284	1.108	0.008	3.980	70.174	10.000
Shapiro-Wilk	0.172	0.350	0.663	0.665	0.887	0.882	0.948
P-value of Shapiro-Wilk	2.348e-14	1.059e-12	1.117e-8	9.262e-9	4.324e-4	3.288e-4	0.048
Minimum	2.203	4.818	0.702	0.016	2.765	25.022	51.000
Maximum	284.165	356.669	10.860	0.111	26.101	639.460	87.000
Fold changes (CTL as reference)	1.04	1.51	1.14	0.91	1.06	1.18	NA

One case in TCP showed extremely high total tau (284.17 pg/dl) and NFL (356.67 pg/dl) and was excluded from the analysis of the rests of total tau.

Figure S1:

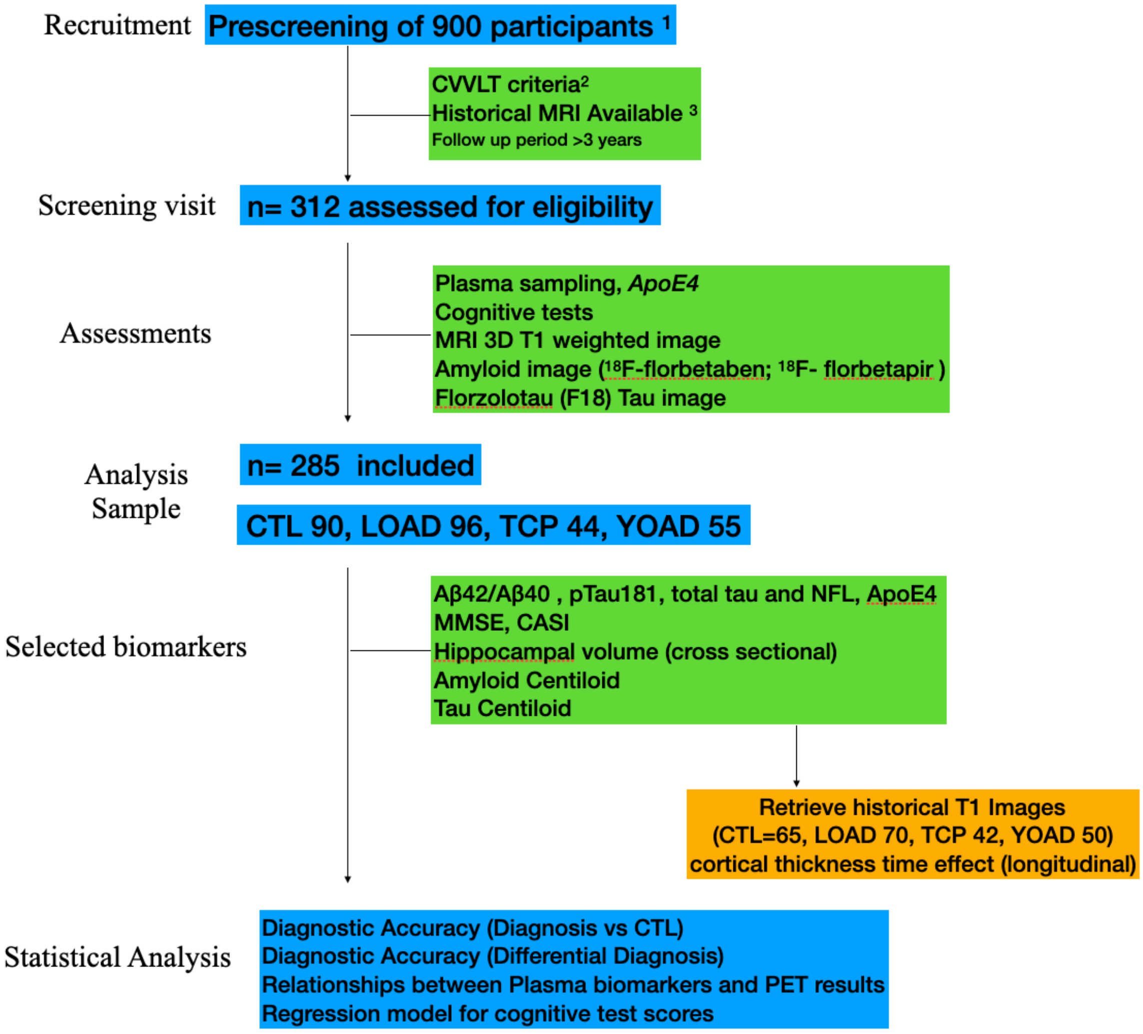

Figure legend: Workflow of this study.
CVVLT: Chinese Version of the Verbal Learning Test; ApoE4: apolipoprotein 4. CTL: cognitively unimpaired controls; LOAD: late-onset Alzheimer's disease; YOAD: young-onset Alzheimer's disease; MMSE: Mini-Mental State Examination; CASI: Cognitive Ability Screening Instrument; NFL: neurofilament light;
In the CTL group, only those >70 years old underwent amyloid and tau imaging (n=30)
[1]Participants enrolled from the Cognitive and Aging Center cohort.
[2]The cutoff CVVLT score for amnestic features or cognitively unimpaired was based on age and educational level (Chang, Kramer et al. 2010).
[3]With available 3DT1 images.

Figure S2

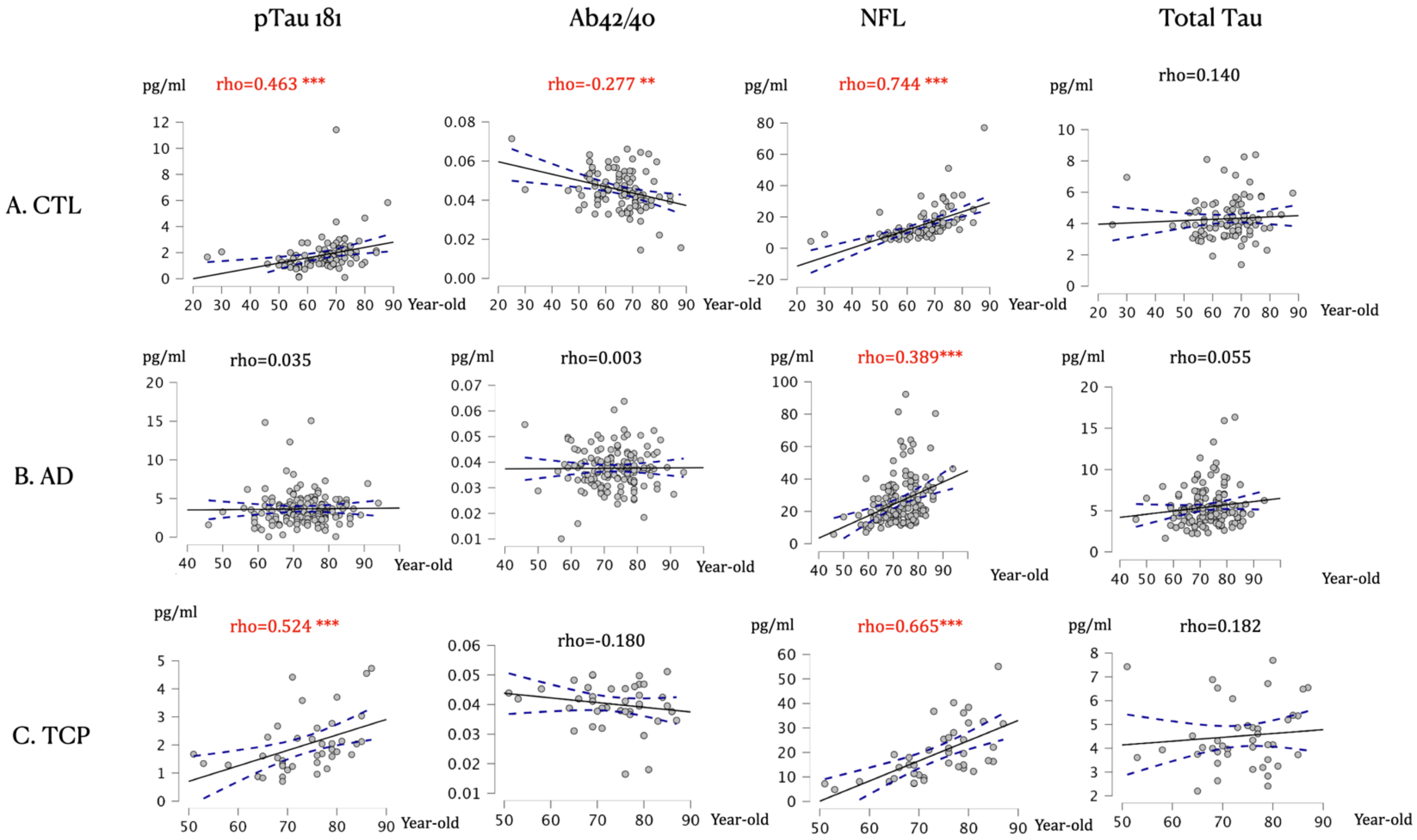

Supplementary Figure 2: Correlation plots with 95% Confidence intervals between levels of plasma biomarkers and age at plasma samplings. The plots are segregated by diagnosis of (A) cognitive unimpaired controls (CTL), (B) Alzheimer's disease (AD), and (C) Tau first cognitive proteinopathy (TCP). Data present as spearman rho correlation coefficient. Significance p value mark as ** $p<0.01$, *** $p<0.005$. NFL: neurofilament light. Outliers with extreme values (listed in Supplementary table 1-3) were excluded.

Figure S3

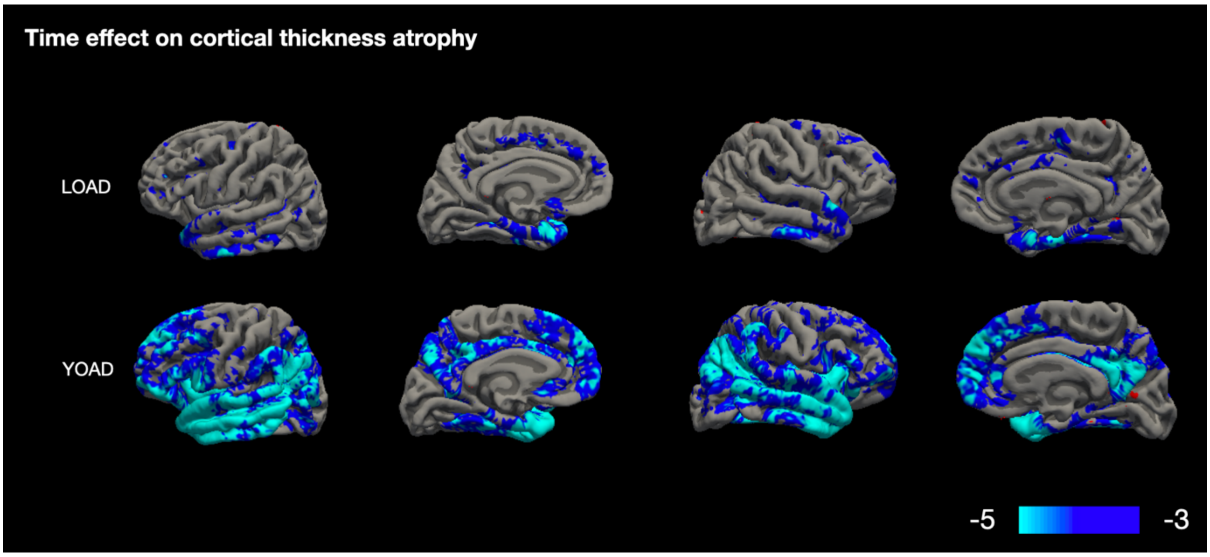

Figure Legend: Time effect modelled using a linear mixed-effect model with longitudinal T1-weighted images. Significance was set as a vertex threshold of 3 with parametric Gaussian-based simulations and cluster-wise corrections. The time effects were only found in the late-onset Alzheimer's disease (LOAD) and young-onset Alzheimer's disease (YOAD) groups were found. Cortical thickness degeneration in the YOAD group was found on topographies of lateral temporal, precuneus and the prefrontal cortex. For LOAD, atrophy was emphasized in the hippocampal and inferior-lateral temporal regions. Cortical thickness time effects were not found in the controls or patients with tau-first cognitive proteinopathy.

Figure S4:

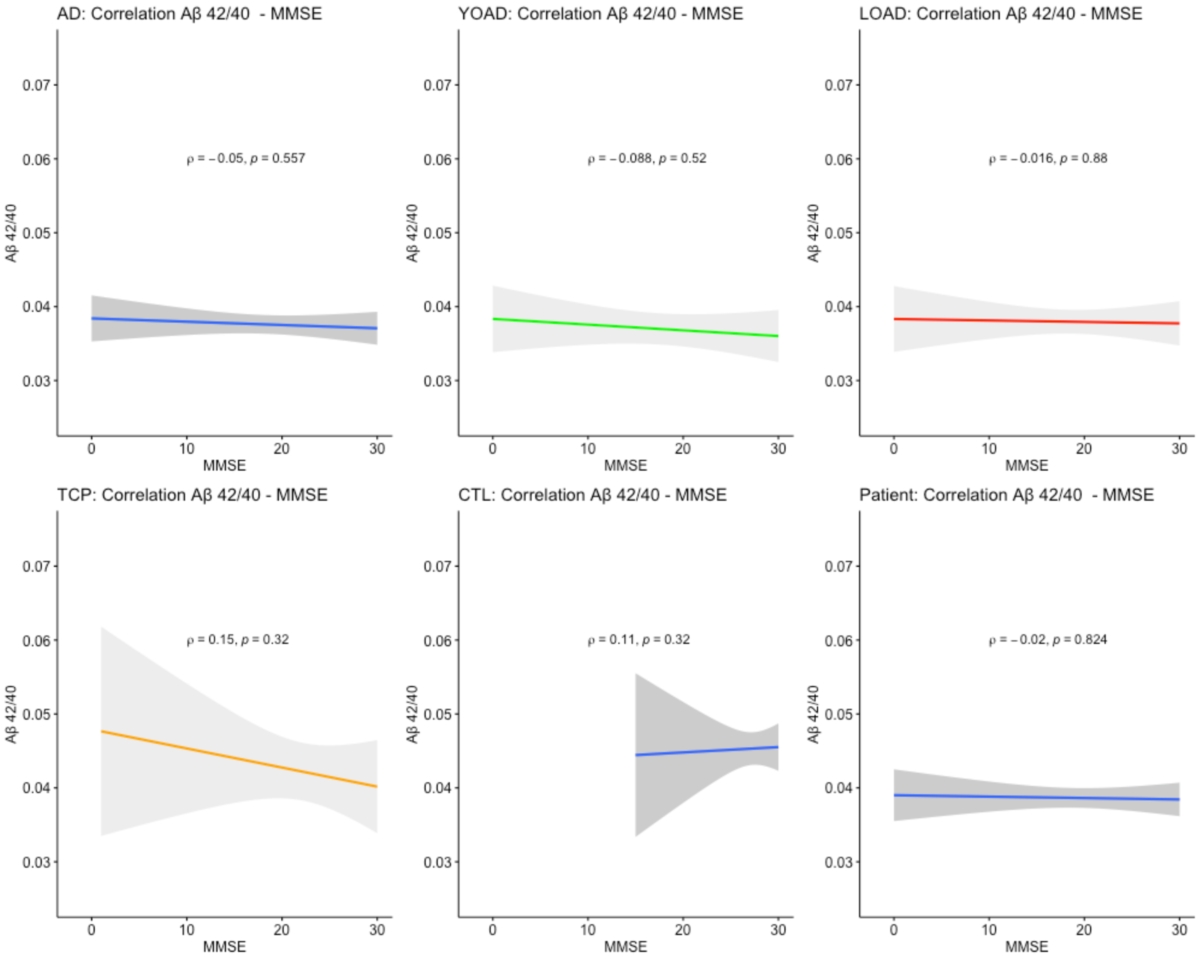

Correlation models of Aβ42/40 ratio on Mini-Mental State Examination (MMSE). None of the regression models showed statistical significance.

AD: Alzheimer's disease; YOAD: young-onset Alzheimer's disease; LOAD: late-onset Alzheimer's disease; TCP: tau-first cognitive proteinopathy. Patient indicates YOAD, LOAD and TCP. CTL: cognitively unimpaired controls

Figure S5:

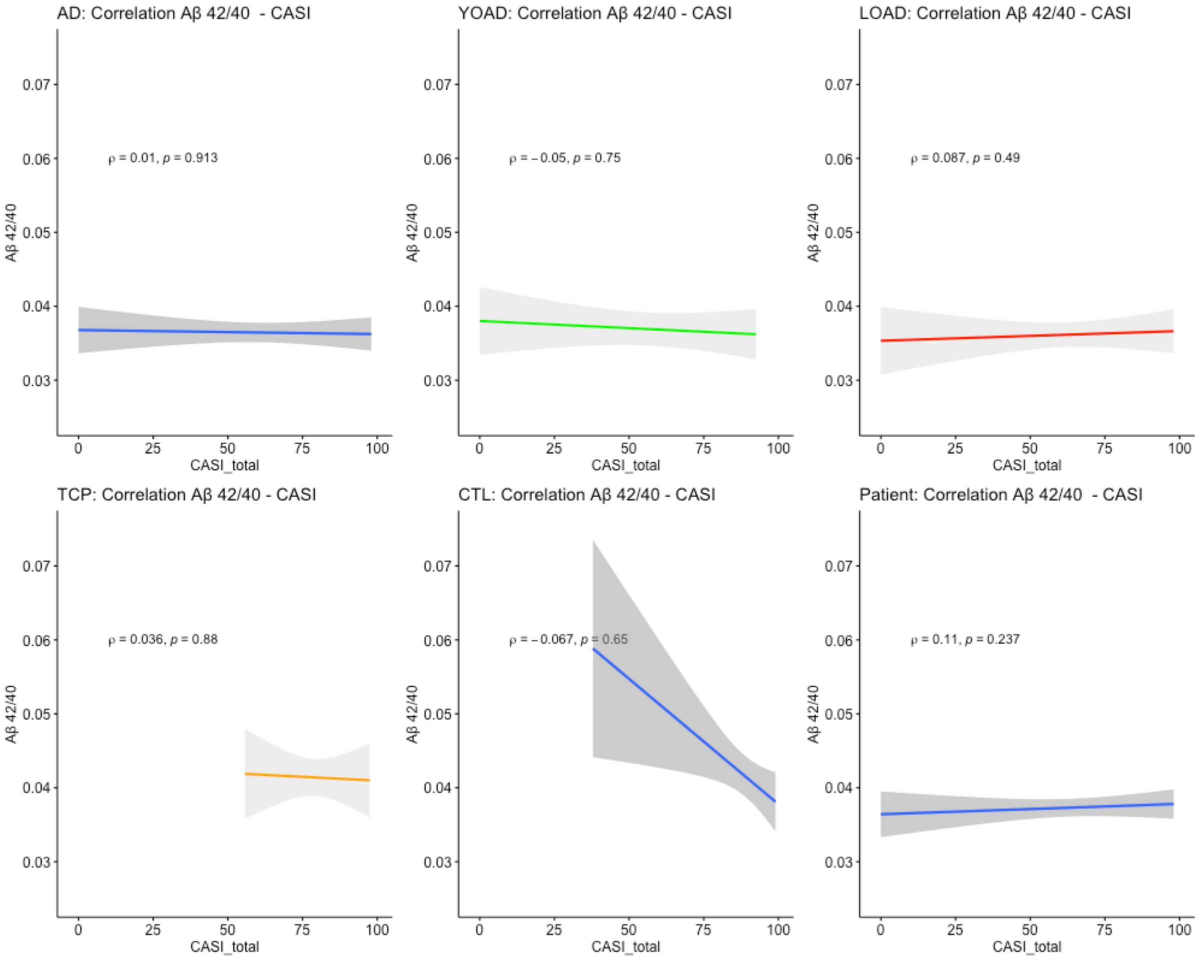

Correlation models of Aβ42/40 ratio on Cognitive Ability Screening Instrument (CASI). None of the regression models showed statistical significance.

AD: Alzheimer's disease; YOAD: young-onset Alzheimer's disease; LOAD: late-onset Alzheimer's disease; TCP: tau-first cognitive proteinopathy. Patient indicates YOAD, LOAD and TCP. CTL: cognitively unimpaired controls

Figure S6:

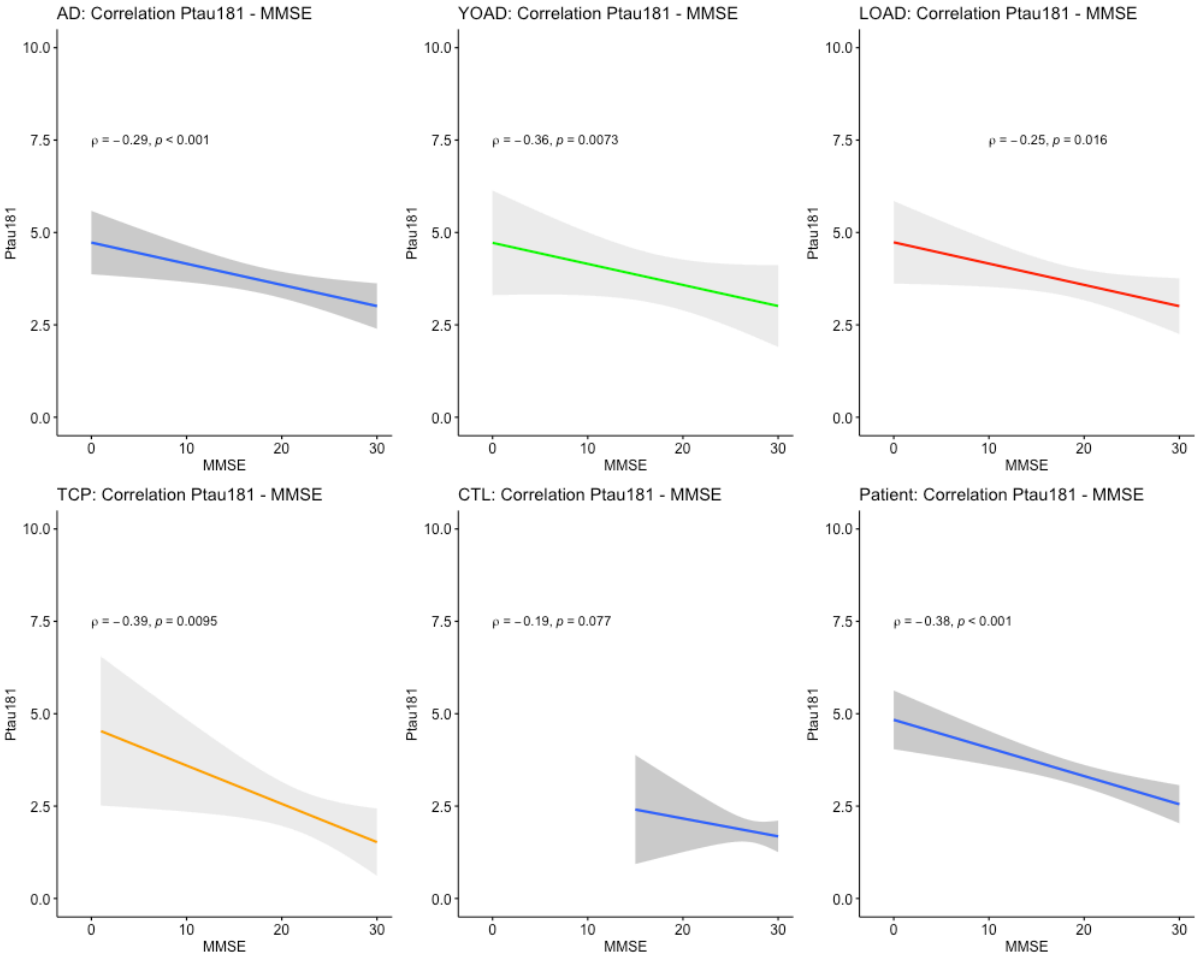

Correlation models of pTau181 on Mini-Mental State Examination (MMSE). The results showed significance in the YOAD, LOAD and TCP groups.
AD: Alzheimer's disease; YOAD: young-onset Alzheimer's disease; LOAD: late-onset Alzheimer's disease; TCP: tau-first cognitive proteinopathy. Patient indicates YOAD, LOAD and TCP. CTL: cognitively unimpaired controls

Figure S7:

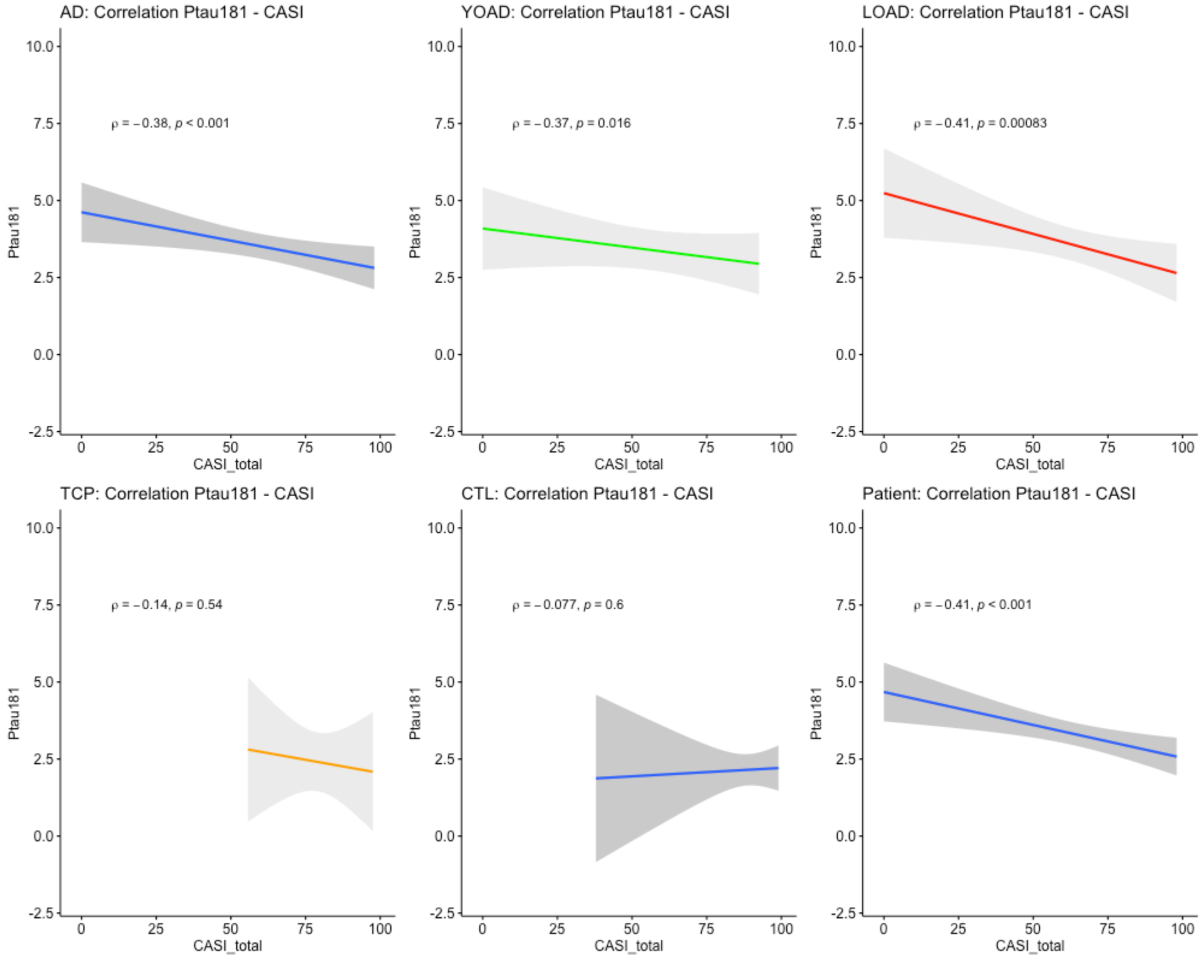

Correlation models of pTau181 on Cognitive Ability Screening Instrument (CASI). The results showed significance in the YOAD and LOAD groups.
AD: Alzheimer's disease; YOAD: young-onset Alzheimer's disease; LOAD: late-onset Alzheimer's disease; TCP: tau-first cognitive proteinopathy. Patient indicates YOAD, LOAD and TCP. CTL: cognitively unimpaired controls

Figure S8:

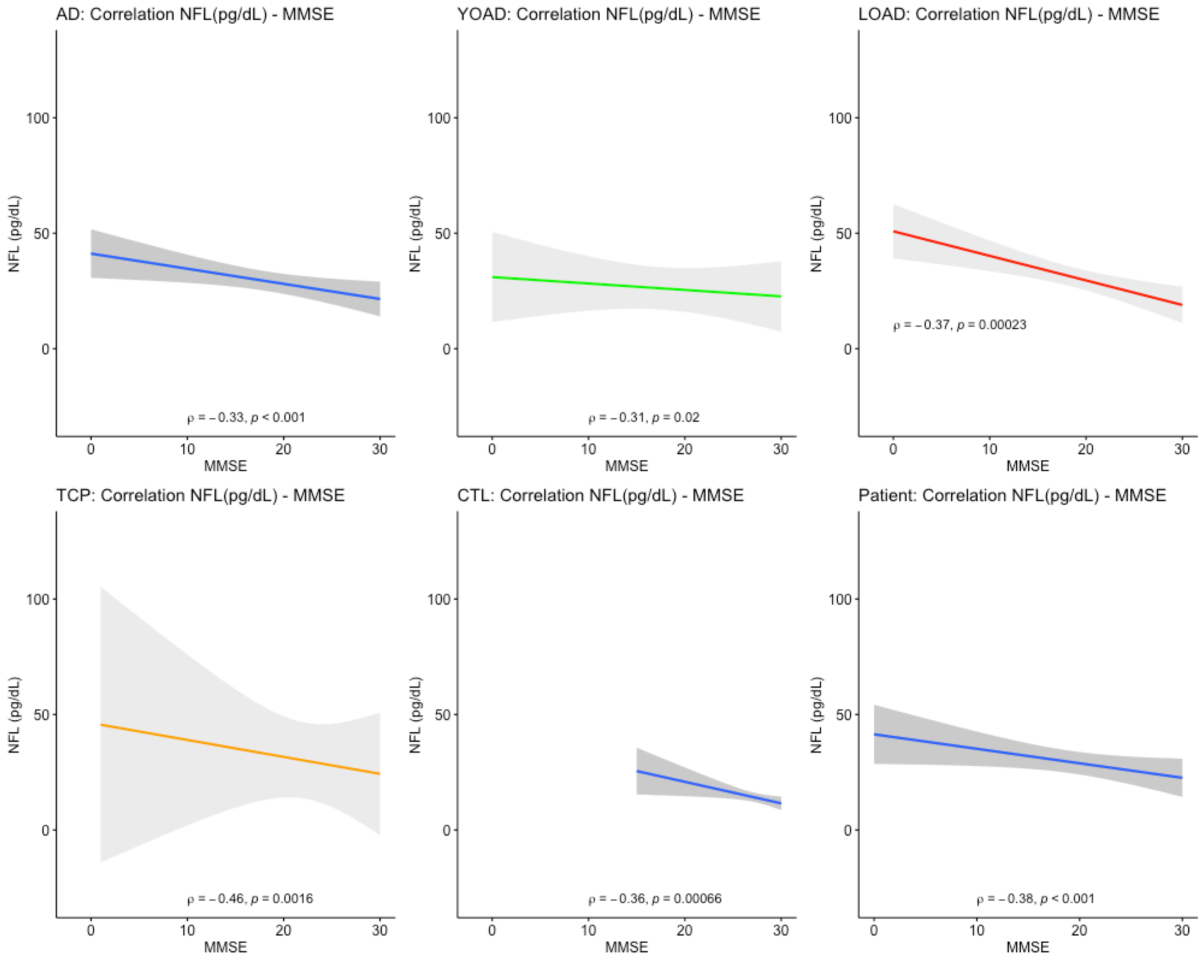

Correlation models of neurofilament light (NFL) on Mini-Mental State Examination (MMSE). The results were significant in all groups.
AD: Alzheimer's disease; YOAD: young-onset Alzheimer's disease; LOAD: late-onset Alzheimer's disease; TCP: tau-first cognitive proteinopathy. Patient indicates YOAD, LOAD and TCP. CTL: cognitively unimpaired controls

Figure S9:

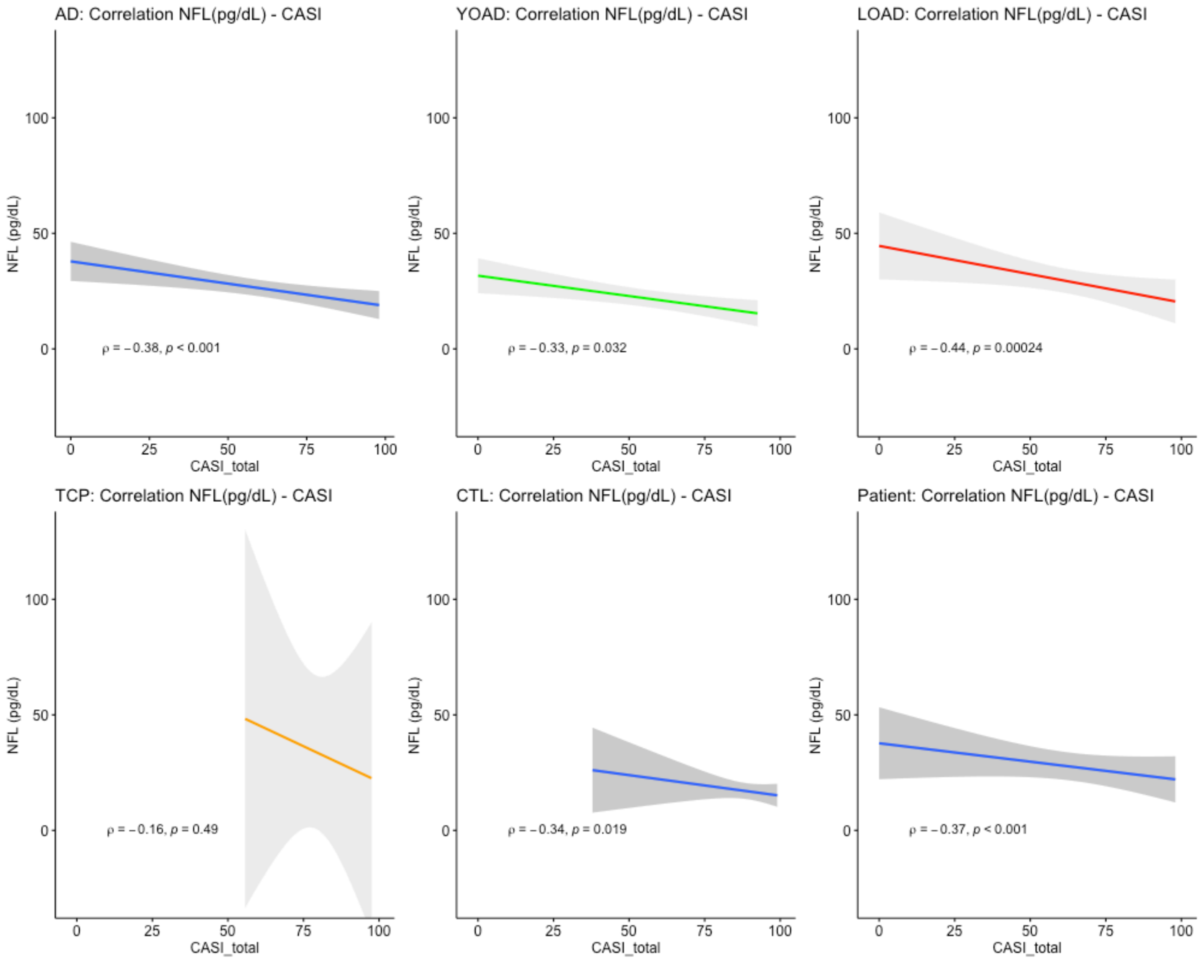

Correlation models of neurofilament light (NFL) on Cognitive Ability Screening Instrument (CASI). The results were significant in the YOAD and LOAD groups.

AD: Alzheimer's disease; YOAD: young-onset Alzheimer's disease; LOAD: late-onset Alzheimer's disease; TCP: tau-first cognitive proteinopathy. Patient indicates YOAD, LOAD and TCP. CTL: cognitively unimpaired controls

Figure S10:

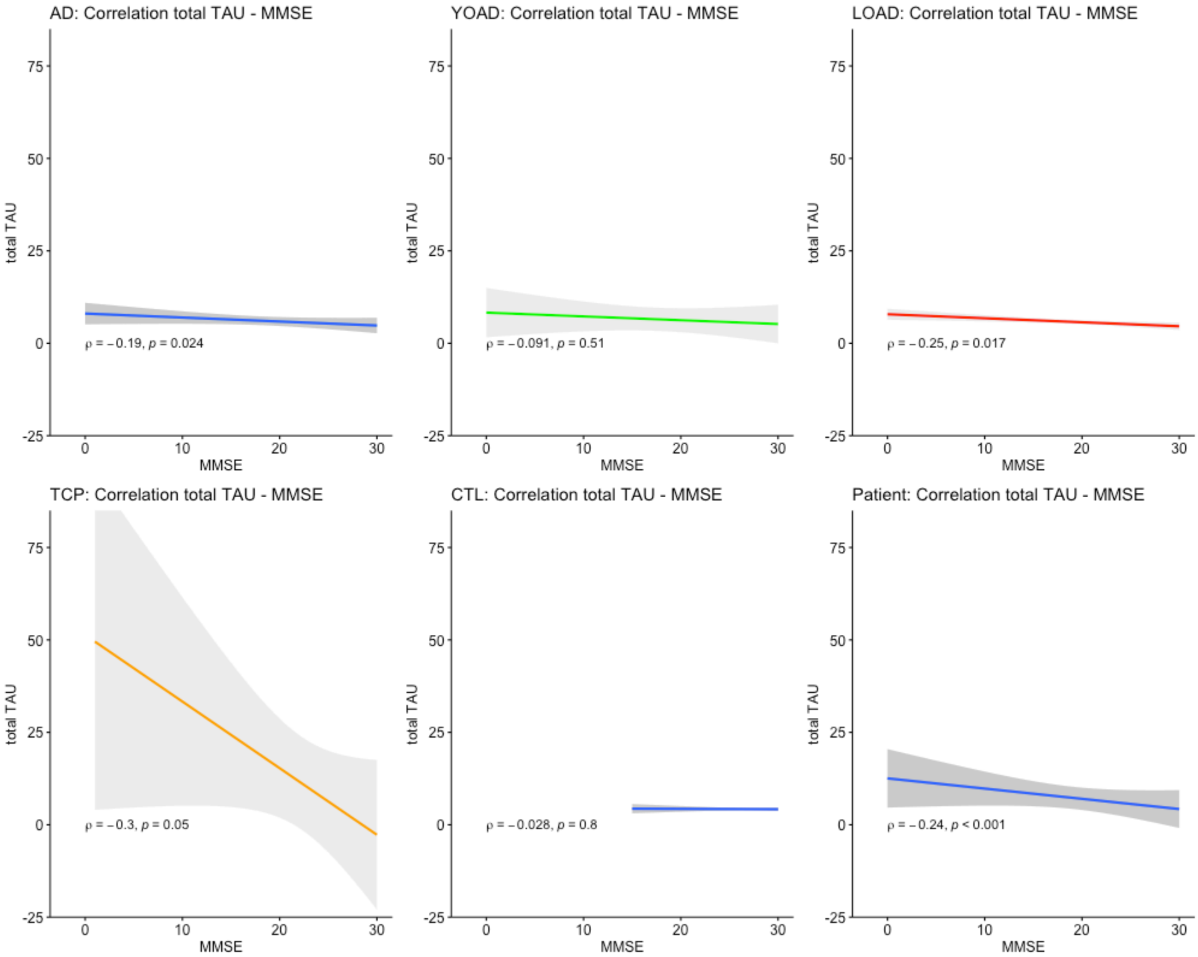

Correlation models of total tau on Mini-Mental State Examination (MMSE). The results were significant in the YOAD and LOAD groups.

AD: Alzheimer's disease; YOAD: young-onset Alzheimer's disease; LOAD: late-onset Alzheimer's disease; TCP: tau-first cognitive proteinopathy. Patient indicates YOAD, LOAD and TCP. CTL: cognitively unimpaired controls

Figure S11:

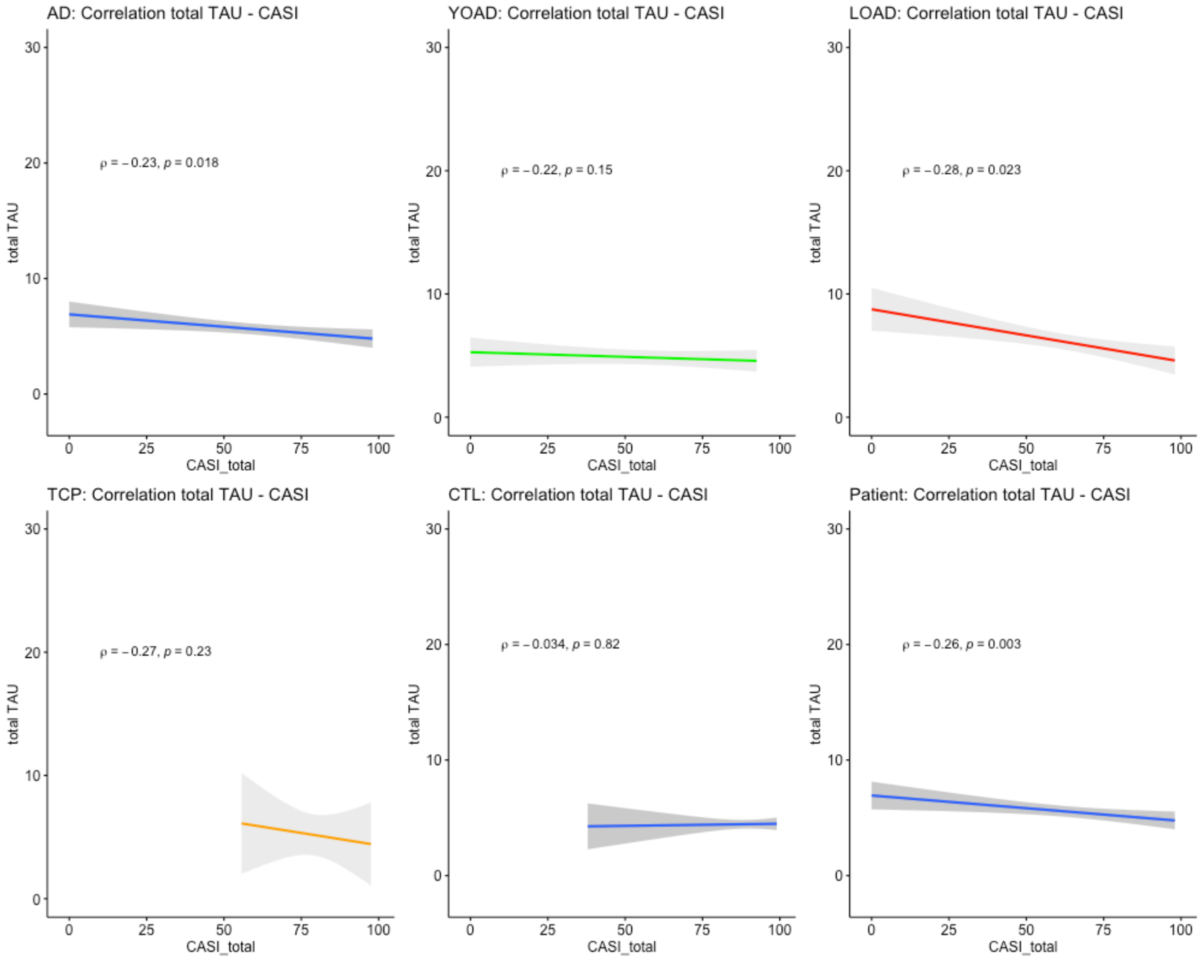

Correlation models of total tau on Cognitive Ability Screening Instrument (CASI). The result was significant in the LOAD group. AD: Alzheimer's disease; YOAD: young-onset Alzheimer's disease; LOAD: late-onset Alzheimer's disease; TCP: tau-first cognitive proteinopathy. Patient indicates YOAD, LOAD and TCP. CTL: cognitively unimpaired controls

Reference:

Chang, C. C., J. H. Kramer, K. N. Lin, W. N. Chang, Y. L. Wang, C. W. Huang, Y. T. Lin, C. Chen and P. N. Wang (2010). "Validating the Chinese version of the Verbal Learning Test for screening Alzheimer's disease." J Int Neuropsychol Soc **16**(2): 244-251.
